# Carotid intima-medial thickness measured on multiple ultrasound frames: evaluation of a DICOM-based software system

**DOI:** 10.1186/1476-7120-5-29

**Published:** 2007-09-24

**Authors:** Kathleen Potter, Daniel J Green, Christopher J Reed, Richard J Woodman, Gerald F Watts, Brendan M McQuillan, Valerie Burke, Graeme J Hankey, Leonard F Arnolda

**Affiliations:** 1 School of Medicine and Pharmacology, University of Western Australia, Perth, Australia; 2 School of Human Movement and Exercise Science, University of Western Australia, Perth, Australia; 3 Cardiac Transplant Unit, Royal Perth Hospital, Perth, Australia; 4 Department of Medical Engineering and Physics, Royal Perth Hospital, Perth, Australia; 5 Department of Medicine, Sir Charles Gairdner Hospital, Perth, Australia; 6 Department of Neurology, Royal Perth Hospital, Perth, Australia; 7 Department of Cardiology, Royal Perth Hospital, Perth, Australia

## Abstract

**Background:**

Carotid intima-media thickness (CIMT) measured by B-mode ultrasonography is a marker of atherosclerosis and is commonly used as an outcome in intervention trials. We have developed DICOM-based software that measures CIMT rapidly on multiple end-diastolic image frames. The aims of this study were to compare the performance of our new software with older bitmap-based CIMT measurement software and to determine whether a ten-fold increase in the number of measurements used to calculate mean CIMT would improve reproducibility.

**Methods:**

Two independent sonographers recorded replicate carotid scans in thirty volunteers and two blinded observers measured CIMT off-line using the new DICOM-based software and older bitmap-based software. A Bland-Altman plot was used to compare CIMT results from the two software programs and t-tests were used to compare analysis times. F-tests were used to compare the co-efficients of variation (CVs) from a standard six-frame measurement protocol with CVs from a sixty-frame measurement protocol. Ordinary least products (OLP) regression was used to test for sonographer and observer biases.

**Results:**

The new DICOM-based software was much faster than older bitmap-based software (average measurement time for one scan 3.4 ± 0.6 minutes versus 8.4 ± 1.8 minutes, p < 0.0001) but CIMT measurements were larger than those made using the alternative software (+0.02 mm, 95%CI 0.01–0.03 mm). The sixty-frame measurement protocol had worse reproducibility than the six-frame protocol (inter-observer CV 5.1% vs 3.5%, p = 0.004) and inter and intra-observer biases were more pronounced in the sixty-frame than the six-frame results.

**Conclusion:**

While the use of DICOM-based software significantly reduced analysis time, a ten-fold increase in the number of measurements used to calculate CIMT did not improve reproducibility. In addition, we found that observer biases caused differences in mean CIMT of a magnitude commonly reported as significant in intervention trials. Our results highlight the importance of good study design with concurrent controls and the need to ensure that no observer drift occurs between baseline and follow-up measurements when CIMT is used to monitor the effect of an intervention.

## Background

Carotid intima-medial thickness (CIMT), measured with B-mode ultrasound, is a marker for atherosclerosis. CIMT is correlated with the known risk factors for cardiovascular disease and is an independent predictor of myocardial infarction and ischemic stroke [1-6]. Consequently, CIMT is used as an outcome measure in intervention trials [7-10] and has recently been promoted as a method for assessing cardiovascular risk in individual patients in clinical practice [11-13].

Edge-detection software has become the accepted standard for CIMT measurement. The use of software improves CIMT reproducibility and reduces observer bias compared with manual techniques [14,15]. Until recently, most software measured CIMT on a single B-mode image frame selected by a sonographer or observer, saved as a bitmap or JPEG image and then opened in the program interface [14,16-19]. In principle, this type of software can be used to measure CIMT on any number of image frames. In practice, the time required to capture, save and load each single bitmap or JPEG image file into the program makes it impractical to estimate CIMT from more than a few measurements per subject.

We have developed software that measures CIMT rapidly on the multiple end-diastolic image frames contained in a single DICOM file. We hypothesize that increasing the number of measurements used to estimate CIMT will reduce random measurement error and improve reproducibility. In addition, DICOM-based software will reduce CIMT measurement time compared with older software by eliminating the requirement to open multiple individual image files. The aims of this study are to compare our new DICOM-based CIMT measurement software with older bitmap-based software and to determine whether a ten-fold increase in the number of image frames used to estimate CIMT improves reproducibility. We have also conducted a systematic review of previously published CIMT reproducibility data.

## Methods

The study protocol was approved by the Royal Perth Hospital Ethics Committee and all subjects gave written informed consent prior to taking part.

### Subjects

30 volunteers chosen to have a range of CIMT values were recruited for the study. Eight subjects were normal healthy volunteers, seven had disordered lipid metabolism and fifteen subjects had a history of stroke or transient ischemic attack. Subject characteristics are presented in Table 1. The subjects attended for two visits separated by one week. Two sonographers scanned each subject in random order on both visits.

**Table 1:** Subject characteristics

	**Subject Type**		
	
	**Normal **(n = 8)	**Hyperlipidaemic **(n = 7)	**Stroke **(n = 15)
Male (n)	2	6	11
Age (years)	38 ± 9	57 ± 11	64 ± 13
BMI (kg/m^2^)	24 ± 3	28 ± 4	30 ± 6
Systolic BP (mmHg)	117 ± 15	135 ± 15	130 ± 15
Diastolic BP (mmHg)	67 ± 8	75 ± 7	72 ± 11
CIMT (mm)	0.54 ± 0.08	0.81 ± 0.26	0.87 ± 0.22

Values are mean ± SD

BMI indicates body mass index; BP, blood pressure; CIMT, carotid intima-medial thickness.

### Ultrasound protocol

The subject lay supine on an examination couch with their head turned 45 degrees away from the side being scanned. The sonographers obtained longitudinal B-mode images of the left and right common carotid arteries, immediately proximal to the carotid bifurcation. Each sonographer adjusted focus and gain settings to optimise far-wall echoes and to minimise noise in the arterial lumen. Dynamic range and image presets, including persistence, edge tracking and pre and post-processing, were not changed between scans. A three lead ECG trace was recorded simultaneously with the B-mode images. The sonographers recorded image sequences for twenty seconds from each of three standardised probe angles (posterior, lateral and anterolateral) in both the left and right common carotids. Each subject thus had four scans (two visits and two sonographers) and each scan consisted six twenty-second image sequences (three from the left carotid and three from the right) stored as a single DICOM file. The 120 scans were recorded to a computer hard-drive using the digital acquisition system described below. A unique four digit ID was used to identify each scan so that observers measuring the CIMT were blinded to subject, visit number and sonographer.

### Image acquisition system

A 10 MHz multi-frequency linear array probe attached to a high-resolution ultrasound machine (Acuson Aspen, Mountain View, CA) was used to record the ultrasound images. A 75Ω coaxial cable from the video output port of the ultrasound machine was plugged into the video input port of a National Instruments IMAQ-PCI-1411, single channel, 8-bit colour image-acquisition board. The analogue video output from the ultrasound machine was converted into a digital DICOM 3.0 file in real time by proprietary DICOM Encoder software and stored on the hard-drive of a standard personal computer running Windows 2000. Image sequences recorded in the DICOM format are stored in a single file as a series of JPEG images that can be viewed individually frame by frame or played as a continuous video sequence. The ultrasound image sequences were acquired at a rate of 25 frames per second and at a resolution of 768 × 576 pixels. A typical scan was two minutes in length, contained approximately 3000 individual image frames and was approximately 250 megabytes in size.

### CIMT analysis software

The CIMT analysis software is written in the icon-based graphical programming language LabVIEW 6.1™ and uses an IMAQ™ vision tool kit for image handling and analysis routines. The software is designed to read DICOM files but can also read single bitmap, TIFF or JPEG image files.

#### ECG detection, frame selection and calibration

**Figure 1:**
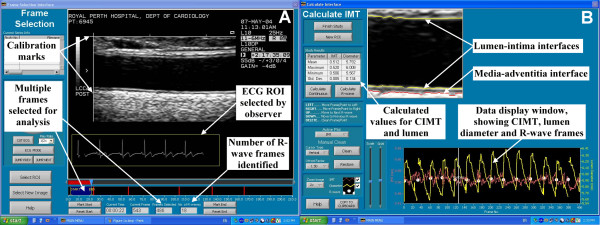
**A: **The frame selection interface of our DICOM-based CIMT measurement software, showing ECG region of interest, ultrasound calibration marks and multiple frame selection indicators. **B: **The calculation interface of our DICOM-based CIMT measurement software showing selected arterial region of interest with lumen and CIMT interfaces marked, data display window and results table.

The observer opens a DICOM file in the analysis software and draws a rectangular region of interest (ROI) around the ECG trace (Figure 1A). An automated R-wave detection algorithm scans along the ECG and records the location of frames co-incident with the R-wave. If the image was not calibrated when the DICOM file was recorded, the observer selects a ROI around the ultrasound calibration marks. The software automatically detects the calibration marks and calculates a pixel-to-centimetre ratio. The observer then selects either a single frame or multiple consecutive frames for analysis. There is no upper limit to the number of frames that can be selected for analysis, other than the length of the recording. When a single frame is analysed, the observer draws a rectangular ROI on the screen that contains both walls of the artery. Multiple frames are selected by marking a start frame and an end frame. CIMT is measured automatically on all the frames between the marked start and end points within a single ROI selected by the user.

#### Edge detection algorithm

The software uses an automated edge-detection algorithm to locate the arterial wall interfaces within the user-selected ROI. The program automatically detects the centre of the artery and divides the ROI into an upper half that contains the near wall lumen-intima interface and a lower half that contains the far wall interfaces. A particle erosion routine is used to condition the image for edge detection by eliminating noise in the lumen of the artery. The threshold used to identify noise is calculated separately for the upper and lower ROI's using a statistical clustering method. The same threshold is also used in the edge detection algorithm. The near-wall intimal edge is initially identified by a Rake routine that scans from the bottom to the top of the upper half of the ROI. The Rake routine uses three parameters of contrast, filter width and steepness to identify and confirm the location of the edge. Contrast is dynamically adjusted for each frame using the calculated threshold value and represents the difference in the average pixel intensity before and after the edge. Filter width specifies the number of pixels averaged either side of the edge and steepness refers to the rate of change of pixel intensity across the edge. The edge is further defined by removing any point outside a pre-set statistical limit.

The same algorithm is used to find the far wall lumen edge, by scanning from the top to bottom of the lower half of the ROI. When the lumen edge has been detected, all the pixels of the intima and media are set to zero (black) and the Rake routine is employed again to find the far wall media-adventitia interface. The edge-detection algorithm identifies the near and far wall lumen edges and the far wall media-adventitia interface on every frame selected for analysis. This process is almost instantaneous for a single frame and takes approximately 10 seconds for 400 consecutive frames.

#### Calculation of CIMT and lumen diameter

When edge detection is complete, the software displays a magnified ROI and marks the lumen margins and the far wall media-adventitia interface with coloured lines (Figure 1B). The software counts the number of pixels in each vertical pixel column between the marked lines and calculates lumen diameter and CIMT using the ratio set during calibration.

If a single frame is selected for analysis, the length of each pixel column is displayed as a data point on a graph below the image. If multiple frames are selected for analysis the software calculates the mean lumen diameter and the mean CIMT for each frame and displays these values as data points on the graphical display. The results from a multiple frame analysis are shown in Figure 1B. The change in mean lumen diameter over repeated cardiac cycles is clearly seen in the graphical display. The mean CIMT values from the R-wave gated frames are identified with white dots. A vertical line on the graphical display indicates which frame the results have come from and the matching image is displayed on the upper half of the screen. The observer can jump from one R-wave gated frame to the next or scroll through all the frames. Erroneous data points are edited by deleting portions of the edge-detection lines from the image or by deleting data points from the graphical display.

When the observer is confident that the three interfaces have been accurately identified on the selected frames, the software calculates the mean, maximum and minimum CIMT and lumen diameter. The results of the calculation are displayed in a table to the left of the image (see Figure 1B). The observer has the choice of measuring these parameters on a single frame, only the R-wave gated frames or on all the frames selected for analysis. The final step in the analysis is the automatic transfer of these results into a MySQL database.

### Comparison of our software against alternative CIMT measurement software

We compared our software with an alternative bitmap-based CIMT measurement software used in previously published studies [20-22]. The alternative software uses a semi-automated edge detection algorithm to measure CIMT on single bitmap images. The user calibrates each image and locates the approximate position of media-adventitia interface with mouse-clicks on the image. The frames and regions of interest selected by one observer for analysis with our software were saved as bitmap files and reanalysed by the same observer using the alternative software. We also compared the time required to measure CIMT using each program.

### Comparison of two different measurement protocols

We tested two different measurement protocols using our new CIMT measurement software: a six-frame measurement protocol and a sixty-frame protocol. The six-frame protocol was based on a standard measurement protocol frequently used in CIMT studies [21,22]. One end-diastolic frame from each of the three left and right carotid image sequences were selected for analysis. CIMT was measured over approximately 10 mm in a section of the artery free of any discrete atherosclerotic plaque on each frame. The final CIMT for each subject was calculated as the mean of these six measurements.

The sixty-frame measurement protocol required the observer to mark the beginning and end frame of a consecutive series of frames from each of the six image sequences. CIMT was measured on a minimum of ten R-wave frames per image sequence and the final CIMT for each subject was thus averaged from at least sixty measurements. The regions of interest for the sixty-frame analysis were selected according to the same criteria as those selected in the six-frame measurement protocol. R-wave gated frames were used in both measurement protocols to eliminate the effect of the cardiac cycle on measured CIMT.

### Sonographer and observer biases

We tested sonographer and observer results for biases using both measurement protocols. The CIMT measurements made by one observer (KP) on all 120 scans were used for the sonographer comparisons. A random sample of 41 scans was selected for the observer comparisons and was analysed twice by each observer with a minimum of one week between first and second analyses.

### Systematic review

We also conducted a systematic review of the literature to allow comparison of our reproducibility results with previous studies. We conducted Medline and Google searches and manually searched citation lists of relevant papers. The keywords we used were *carotid intima medial thickness *or *IMT *or *intima media *and *validation *or *reproducibility *or *software *or *edge-tracking*. We also searched for reproducibility statistics in the method sections of papers reporting CIMT as an outcome. We included all studies that reported inter- and intra-sonographer and observer CVs and calculated CVs for other papers when the appropriate data was given (mean CIMT and SD of absolute differences).

### Statistical analysis

A Bland-Altman plot was used to compare the new software with alternative CIMT measurement software. We used a paired t-test to compare the mean CIMT measurement time for each program. The co-efficients of variation (CVs) from a standard six-frame measurement protocol were compared with CVs from the sixty-frame measurement protocol using F-tests. Ordinary least products (OLP) regression was used to test for sonographer and observer biases. We chose OLP regression to test for bias as it allows for both the x and y values to be attended by random error and thus requires no judgement about whether the y variable or the x variable provides 'true' values [23]. It is thus particularly useful in reproducibility studies as it provides a technique for calibrating one method or measurer against another when neither is a gold standard. All calculations were performed using the technique described by Ludbrook [24].

## Results

### Comparison of our CIMT measurement software with alternative software

**Figure 2:**
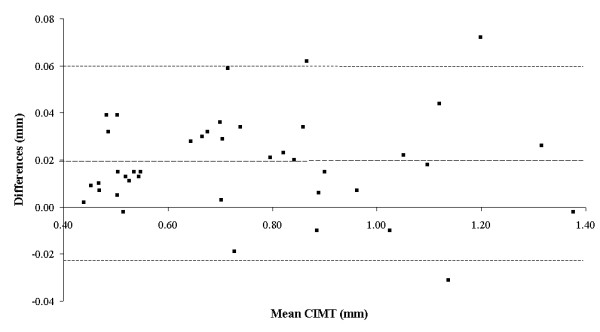
Bland-Altman plot of the differences between the CIMT measured with our new DICOM-based software and the alternative bitmap-based software. Differences are calculated as our software minus the alternative software and are plotted against the mean CIMT (mm) from both software programs. Dashed lines show mean ± 2SD for estimated difference between software programs.

Figure 2 shows the differences between the CIMT measurements made using our software and the alternative software program plotted against the mean CIMT from both programs. The mean difference was 0.019 mm (95%CI 0.012 to 0.025 mm). The upper limit of agreement was 0.060 mm (95%CI 0.049 to 0.072 mm) and the lower limit was -0.023 mm (95%CI -0.033 to -0.011 mm). On the recorded images, one pixel represents approximately 0.05 mm. It took 3 minutes and 22 seconds (SD 34 seconds) to measure CIMT on six frames using our software and 8 minutes and 24 seconds (SD 109 seconds) with the alternative software (t = -8.31, df = 80, p < 0.0001).

### Comparison of two different measurement protocols

**Figure 3:**
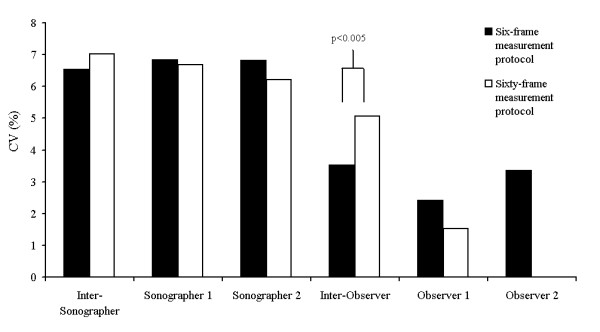
Comparison of the coefficients of variation for CIMT measured with our DICOM-based software using a six frame measurement protocol and a sixty-frame measurement protocol.

The sixty-frame measurement protocol did not significantly improve reproducibility compared with the standard six-frame protocol (Figure 3). An apparent improvement in the first observer's intra-observer CV was not significant (1.5% vs 2.4%, F = 0.76, df = 37, p = 0.20) and the sixty-frame inter-observer CV was significantly worse than the six-frame CV (5.1% vs 3.5%, F = 0.41, df = 36, p = 0.004). It took 11 minutesand 8 seconds (SD 120 seconds) to measure CIMT on sixty frames.

### Sonographer and observer biases

Table 2 shows the results of OLP regression testing for sonographer and observer biases. No inter or intra-sonographer biases were present in results from either measurement protocol. Fixed and proportional biases were present in the observer comparisons from both protocols, but were more frequent and more pronounced in the sixty-frame results.

**Table 2:** OLP regression results for CIMT measurements

**Comparison**	**a'**	**95% CI**	**b'**	**95% CI**	**Fixed Bias**	**Proportional Bias**
*Six-frame measurement protocol*						
Inter-Sonographer	0.048	-0.03, 0.12	0.964	0.87, 1.07	No	No
Intra-Sonographer 1	0.013	-0.08, 0.10	1.002	0.89, 1.13	No	No
Intra-Sonographer 2	-0.025	-0.12, 0.06	1.025	0.92, 1.15	No	No
Inter-Observer	-0.024	-0.06, 0.01	1.053	1.01, 1.10	No	Yes
Intra-Observer 1	0.004	-0.02, 0.03	0.992	0.96, 1.03	No	No
Intra-Observer 2	-0.031	-0.07, 0.003	1.056	1.01, 1.11	No	Yes
*Sixty-frame measurement protocol*						
Inter-Sonographer	0.001	-0.10, 0.09	1.027	0.91, 1.16	No	No
Intra-Sonographer 1	-0.008	-0.11, 0.08	1.017	0.90, 1.15	No	No
Intra-Sonographer 2	-0.009	-0.10, 0.07	0.995	0.89,1.11	No	No
Inter-Observer	-0.106	-0.17, -0.05	1.159	1.08,1.24	Yes	Yes
Intra-Observer 1	-0.020	-0.04, -0.003	1.031	1.01,1.05	Yes	Yes

OLP indicates ordinary least products; CIMT, carotid intima-medial thickness; CI, confidence interval.

The ordinary least products regression equation is y = a' + b'x. 95% CIs are for the population parameters α ' and β '. Fixed bias is present if the 95% CI for α ' does not contain zero. Proportional bias is present if 95% CI for β ' does not contain one.

### Systematic review

We identified twenty-eight published studies that reported suitable reproducibility data for CIMT measurement. The CVs from these studies, along with data from the current project, are presented in Table 3, divided according to the method used to measure CIMT.

**Table 3:** Coefficients of variation from CIMT reproducibility studies

		**Coefficient of Variation**			
		
**Study**	**n**	**Inter-sonographer**	**Intra-sonographer**	**Inter-observer**	**Intra-observer**
*Manual Measurement (callipers on ultrasound screen)*					
Salonen^a ^[27]	10	10.5	5.4	-	-
Bonithon-Kopp^b ^[35]	81	-	-	9.0	-
Schillaci^c ^[36]	128	-	-	7.3	5.0
Raitakari [37]	60/113	-	6.4	5.2	-
Kobayashi [38]	12	-	4.2	-	-
*Software 1 (arterial interfaces traced manually by observer)*					
Wendelhag [39]	74	10.2	-	-	-
Bots^b ^[40]	80	8.1	-	4.5	-
Persson [41]	43	11.4	8.0	-	-
Touboul^b ^[16]	14	12.5	8.6	-	-
Riley^a, b, c ^[42]	453	7.6	6.1	6.8	3.8
Wendelhag [14, 43]	50	-	10.6	2.8	3.8
Liang [44]	50	-	2.8	-	-
Nowak [45]	-	-	9.0	-	-
Baldassarre [46]	22	-	4.1	-	-
Baldassarre^d ^[46]	22	-	2.1	-	-
Becker [47]	7	-	11.0	-	-
Oren^b, c ^[48]	21	5.4	-	-	-
*Software 2 (automated edge-detection)*					
Selzer [18]	8	3.9	4.3	-	-
Blankenhorn [49]	20	-	-	3.1	3.0
Gariepy [17]	11	-	4.3	-	-
Adams [20]	35/100	6.0	-	2.5	-
Wendelhag [14]	50	-	-	1.4	-
Stensland-Bugge [26]	75	9.0	5.9	-	1.3
McQuillan [22]	30	5.9	2.9	-	-
Selzer [28]	24	-	3.5		
Kennedy^c ^[50]	144	-	-	6.8	6.7
Fathi [51]	288	-	-	-	5.0
Gepner [12]^e^	40	-	-	-	3.1
Gepner [12]^f^	40	-	-	-	7.8
de Bree [52]	80	6.5	-	-	-
Potter	30	6.8	5.7	-	2.1
*Software 3 (current study)*					
Potter^g^	30	6.5	6.8	3.5	2.4
Potter^h^	30	7.0	6.2	5.1	1.5

CV indicates co-efficient of variation; CIMT, carotid intima-media thickness; CVD, cardiovascular disease; CCA, common carotid artery.

^a ^Maximum rather than mean CCA used for reproducibility study

^b ^CVs calculated from reported data (SD of absolute difference/mean CIMT *100)

^c^CIMT calculated as average of near and far wall of CCA

^d ^Digital ultrasound system

^e^Experienced observer

^f^Novice observer

^g^Six-frame protocol

^h^Sixty frame measurement protocol

## Discussion

This study has produced several results of interest to investigators using CIMT as a marker of cardiovascular risk. Our new DICOM-based software halved CIMT measurement time compared with an alternative system designed to read bitmap files. However, increasing the number of measurements used to estimate CIMT did not improve reproducibility and measuring CIMT on sixty-frames appeared to increase the risk of bias within and between observers compared with measuring CIMT on fewer frames.

The strengths of this study were that we validated our software in subjects with a wide range of CIMT values and tested it against an alternative software program. We used blinded observers and the study was designed so we could measure sonographer and observer reproducibility separately. We also conducted a systematic review of previously published CIMT reproducibility data. A limitation of this study was that one observer did not perform replicate measures with the multiple-frame protocol, reducing our ability to detect improvements in intra-observer reproducibility.

The CIMT values from our software were approximately half a pixel larger than CIMT values from the alternative software (+0.02 mm, 95%CI 0.01 to 0.03 mm) with evidence of an increase in the size of the differences for larger CIMT values (Figure 2). The same frames and regions of interest were used with both software programs, so differences in the edge-tracking algorithms probably caused the detected biases. It is also possible that observer editing contributed to the discrepancy between programs, but is more likely to account for the proportional than the fixed bias. Our results show that different edge-tracking algorithms will produce different CIMT values for the same recorded images, something that should be kept in mind if absolute CIMT values are ever compared between studies.

The sonographer reproducibility results in this study are similar to those reported by previous investigators (Table 3). We tested sonographer reproducibility by comparing CIMT measured by one observer on two separate scans of the same subject recorded by the same or different sonographers. Our finding that a sixty-frame measurement protocol did not improve intra- or inter-sonographer reproducibility was not altogether unexpected, as previous reports show that changes in subject positioning, transducer angle and ultrasound settings account for the greatest proportion of between-scan variation in CIMT [25-27]. Although we used a standardised scanning protocol to reduce between-scan differences, increasing in the number of measurements on each recorded scan did not significantly reduce the variation in CIMT caused by sonographer differences. Investigators who used a hard copy of each subject's baseline image to match the repeat scan to the previously recorded image found that averaging measurements from five frames reduced intra-sonographer variability relative to single frame measurements [28]. Their results suggest that, if sonographer differences could be sufficiently reduced by such a technique, multiple measurements might further improve between-scan reproducibility.

By contrast with sonographer reproducibility, we had expected the sixty-frame measurement protocol to improve observer reproducibility. Observer comparisons were made on replicate analyses of the same scan, eliminating the between-scan variation in CIMT due to sonographer differences. Increasing the number of measurements used to calculate CIMT should have reduced random measurement error and improved both inter and intra-observer reproducibility. Although we found a small reduction in the sixty-frame intra-observer CV, it was not statistically significant and the inter-observer CV was significantly worse than the corresponding six-frame CV.

Bias within and between observers was probably responsible for the lack of improvement in reproducibility with the sixty-frame measurement protocol. Inter and intra-observer drift is a recurrent problem in large trials that use CIMT as an outcome [29-31]. In the current study, we found that one observer's CIMT measurements were proportionally greater than the other's measurements on the same scans. In addition, both observers' first measurements differed systematically from their second measurements on the same scans (Table 2). For these biases to have occurred, observers must have changed their frame selection and/or choice of ROI and/or their editing of the detected interfaces between analyses. Instead of reducing random measurement error as intended, the sixty-frame measurement protocol magnified these differences, causing more pronounced bias and worse inter-observer reproducibility. Although we used a protocol to reduce systematic differences between sonographers, we had assumed that automated edge-detection would prevent observer biases and thus spent insufficient time prior to the study ensuring that our observers were producing consistent results.

To give the OLP regression results some context, the bias within and between observers resulted in a difference of approximately one pixel (or 0.05 mm) in measured CIMT. Although this difference may seem trivial, it should be noted that CIMT progresses very slowly in most people, at the rate of 0.001 mm to 0.03 mm per year [32,33]. Reductions in mean CIMT of a magnitude smaller than the between-observer biases detected in this study are frequently reported as significant in intervention trials [2]. Our results have obvious implications for the design of clinical trials, reinforcing the importance of concurrent controls and careful monitoring of observer drift in CIMT measurements, particularly if scans are read at multiple centres or at several time points during the trial. The recent development of portable digital ultrasound machines and user-friendly measurement software means that CIMT is often promoted as a method for assessing cardiovascular risk or the effect of therapeutic interventions in individual patients [11,34]. Clinicians who intend to monitor CIMT in individual patients should be aware that between-scan differences due to measurement error may be of greater magnitude than any biological variation.

## Conclusion

While the use of DICOM-based software significantly reduced analysis time, a ten-fold increase in the number of measurements used to calculate CIMT did not improve reproducibility. In addition, we found that observer biases caused differences in mean CIMT of a magnitude commonly reported as significant in intervention trials. Our results highlight the importance of good study design with concurrent controls and the need to ensure that no observer drift occurs between baseline and follow-up measurements when CIMT is used to monitor the effect of an intervention.

## Competing interests

The author(s) declare that they have no competing interests.

## Authors' contributions

KP was participated in the design of the study, recorded the ultrasound scans, made the CIMT measurements, wrote the manuscript and performed the statistical analysis. DJG participated in the design of the study, obtained funding for the software development, and critically reviewed the manuscript. CJR wrote and developed the software, gave technical assistance and critically reviewed the manuscript. RFW recorded the ultrasound scans, assisted with interpretation of the data and statistical analysis, and assisted with manuscript preparation. GFW participated in the design of the study, obtained funding for the software and critically reviewed the manuscript. BMM participated in the design of the study and critically reviewed the manuscript. VB participated in the design of the study, assisted with statistical analysis and critically reviewed the manuscript. GJH critically reviewed the manuscript. LFA participated in the design of the study and critically reviewed the manuscript. All authors read and approved the final manuscript.
